# Southern Dental Association

**Published:** 1896-02

**Authors:** Jennie M. Walker

**Affiliations:** Bay St. Louis, Miss.


### Southern Dental Association.

REPORTED BY MRS. JENNIE M. WALKER, BAY ST. LOUIS, MISS.

The Southern Dental Association held its twenty-sixth annual meeting at Atlanta, Georgia, November 5th, 6th, 7th and 8 h, 1895.

The meeting was called to order at 11 A. M., with the President, Dr. H. E. Beach, of Clarksville, Tennessee, in the chair.

After prayer by the Rev. I. B. Robbins, and a brief address of welcome from Dr. Frank Holland, of Atlanta, the President read his annual address. He drew a beautiful parallel between the gigantic strides of material progress made by Atlanta during the past twenty-six years, and the equally wonderful strides in scientific advancement made by the Southern Dental Association during the same period. The leading thought in the address was the importance of seeking, in the discussion, to reach definite conclusions that could and would go out as authority. The people, as they become better educated in regard to dentistry, demand a reason for what we do, and if we could give the conclusions that have been reached after full and free discussions in this body, it would be an authority of the highest order, especially in the settlement of legal questions. He suggested, therefore, that some plan be agreed upon by which the conclusions arrived at might be formulated and recorded in a manner easy of reference. This he considered especially necessary in regard to the demonstrations made in the clinics, in order that the Association might either endorse or reject the demonstrations. One advantage in the latter regard would be to do away with the unprofessional *advertising* sometimes done through clinics. Matters deemed of sufficient importance, presented in the clinics, might be referred to an appropriate committee for investigation, to be reported upon subsquently, thus enabling intelligent action on the same.

He spoke of the lamentable facts, admitted by every one who has been through a dental college, that every class has its class theories—that it is not safe to leave anything worth having, laying around where it can be appropriated. If a student will steal instruments and materials from his classmates, what may not be expected from him in his dealings with his patients and with the public? Though he may be educated mentally to the highest possible standard, morally he will be the same. Means should be devised by which the *good* might be weeded out from the *bad*, before they are licensed to practice their evil desires for gain upon a trusting public. Let not the colleges give to the profession men that are untrustworthy, then will a new era begin to dawn.

In view of the expressed determination of the President of the American Association, that, "even if the Southern Association does not come in, we will make the *American* Dental Association thoroughly and genuinely, not only in name but practically, the society that shall pass upon all questions of ethics, and other questions that should be adjudicated by the whole profession" (Cos. May, '95, p. 447), he said that admitting as a condition greatly to be desired that there should be an organized body of American dentists, qualified and having the authority to act officially in such matters, he could not agree that either the "American" or the "Southern" or any other Association as now organized should assume to itself the prerogative of legislating or prescribing rules to govern the profession at large. He then proceeded to outline a national organization which should be to the dental profession what the United States Senate is to the American citizen—the membership to be representative through election by State Societies in proportion to the number of registered dentists in the State; such a body to meet annually, being elected, with authority to formulate rules ethical, legislative and judicial, and to adjudicate all questions referred to it concerning the dental profession. The members of such an organization should be elected for one year and be eligible for re-election as long as their actions were approved by their constituency. Such a body of men, coming fresh from every section of the country, would be better qualified to represent the wants and necessities of the whole profession than any organization composed of a membership whose sole qualification is the payment of his dues.

The American Association having appointed a committee of five to confer with a similar committee for the Southern Association with a view to the consolidation of the two Associations, the President recommended the appointment of such a committee.

The President's address was referred to a committee of three, with instructions to report back to the Association the points deemed most worthy of discussion.

After a reading of the minutes of the last annual meeting by the Secretary, Dr. S. W. Foster, and roll-call and collection of dues, the Association adjourned to 3 o'clock P. M., when the report of the committee on Operative Dentistry was called for. The Chairman not being present, the subject was passed for the time being, and report of committee on Prosthetic Dentistry called for. The Chairman, Dr. R. K. Luckie, announced a paper by Dr. W. E. Walker, of Pass Christian, Mississippi. Dr. Walker then read a paper entitled: "The Glenoid Fossa; the Movements of the Mandible; the Cusps of the Teeth," of which we give a synopsis:

Without repeating what is found "in the books" on these topics, he referred to the statement generally made, that the movement of the condyle in the fossa is invariably described as "forward." For reasons to be given he was led to close observation on this point, and found that the movement is really a combined "forward and downward" movement, due to the incline or slant of the roof of the glenoid fossa of the condyle, both in its anterior and lateral excursions, traversing a course forming an angle on an average of about 35° to a line perpendicular to the facial line, or of about 55° to the facial line. The condyle, not swinging directly from its upper surface, but as though pivoted in an imaginary slot about fifteen millimeters above its upper surface, all of the condyle above this point moving forward, while all below it (the ramus) swings back, in opening the mouth, the peculiar articulation of the mandible gives it a back and forward rocking motion, and also a swinging or see-sawing motion from side to side in taking the position affording incising and the grinding articulation of the teeth in mastication. These movements, as also the articulation of the teeth, cannot be studied from the naked skull, as the glenoid fossa is then emptied of its intro-articular fibro-cartilage, its double synovial sacs and its membranes, leaving a space between the upper surface of the condyle and the roof of the glenoid fossa, so that when the condyle is thrown up in the socket the teeth fail to articulate, though they occlude correctly when in the position of rest. These movements must be studied either from the living subject (selecting as their accommodating subject with no superfluity of flesh), or from a properly conducted articulator, which shall permit a perfect reproduction of all the movements of the mandible, including the "downward and forward" course traversed by the condyle, with the consequent drop of the ramus, influencing the articulation of the teeth in the varied positions of the jaw.

The movement of the condyle constitutes an important factor to be borne in mind in studies of the articulation of the teeth, as for the shaping of artificial cusps, whether in filling, crown-work, bridge-work or plate-work; in the study of the natural cusps, with a view to bettering the articulation, both in the treatment of pyorrhœa alveolaris, and in orthodontia; in the study of diseases of the facial muscles, or of the glenoid fossa, etc.

He said that he was first led to the study of the subjects named in the title of his paper from meeting with unsatisfactory results in the use of the Bonwill articulator, articulating artificial teeth by the rules laid down by Dr. Bonwill, the articulation of the teeth in the mouth not being the same as in the Bonwill articulator, the occlusion, however, being as perfect and the same in the mouth as in the articulator.

Seeking the cause for this difference in articulation, he began the study of casts or models of the natural teeth placed in the Bonwill articulator, finding that while occlusion would be perfect, he could not reproduce the normal points of contact of the cusps in the different portions of the jaw as found in the mouth from which the casts were taken. Having verified this observation from the study of casts of a large number of as nearly normal teeth as we meet with, studying the articulation of the cusps, both in the mouth and from the casts in the Bonwill articulator, careful experimentation showed that the right angle found in that articulator at the junction of the parts representing the ramus, and the condyle moving in the glenoid fossa, was the cause of the defective articulation; a Bonwill articulator remodeled in such a manner as to raise the portion carrying the spiral springs until the angle was increased an average of thirty-five degrees, permitting the correct articulation of the teeth of a large number of models, in any position in which the lower jaw, either of the mouth or of the articulator, can be placed, whether as for "biting" or as for "grinding," either on the right side or the left. Artificial teeth having the cusps modeled, or the natural teeth so remodeled, would also articulate correctly in the mouth.

The articulator so remodeled he christened the "Walker-Bonwill" articulator.

Further experiments showed, however, that it was necessary to have *an adjustable angle*, to meet the variations from the average (or what might be termed *the normal*) ranging from 30° to 45°; an extreme case of irregularity registering as low as $22\frac{1}{2}$°.

The articulator constructed with the adjustable angle, with set screws to secure it and gauges to register the degree of the angle found in individual cases, which sometimes varies even in the two sides of the same face, with a further modification enabling the correction of "a wrong bite," constitutes what we have called the "Walker Physiological Articulator," because it is not only automatically but also physiologically (that is functionally) correct throughout.

Seeking the cause of the peculiar features in the articulation of models of the natural teeth, in the movements described by the lower jaw of the reconstructed articulator, led to the discovery of the *downward* as well as "forward" movement of the condyle in the antero and lateral excursions of the mandible, and also in opening the mouth; which, so far as he has been able to ascertain, has hitherto escaped observation, or perhaps not been deemed of sufficient importance to be placed on record in the literature of human anatomy. Its practical importance to the dental specialist has been indicated.

To reproduce with artificial teeth the articulation of the natural teeth, in order to give the grinding and biting functions to artificial dentures, instead of the usual up-and-down *mashing* action of full dentures with nearly cuspless teeth, led to the minute study of the cusps of the human teeth, both in the mouth and from models and their inter-articulation. This led to the discovery of what might be called the law of the cusps, the variation in the distance from the base of the sulcus to the point of the cusp from the main lower cusps of the second bicuspid increasing distally in the superior lingual cusps and the inferior buccal cusps, conversely decreasing distally in the superior buccal cusps and the inferior lingual cusps.

This is most clearly seen by placing the model of as perfect a set of natural teeth as can be obtained, *cusps downward*, on a clear slab of glass, bringing the successive pairs of teeth under observation to the edge of the glass, where the relative height of the cusps will, as a rule, be found as stated.

A chart was exhibited showing Dr. Bonwill's rear view of the articulation of the molars in the masticating position, with *all the cusps*, upper and lower, buccal and lingual, *on the same plane*, as found in Harris' Principles and Practice, 12th ed.—and also as reproduced in the chart found in Trans. W. C. D. Congress, 1893—all showing the relative position of the same level-cusp lower molars in the normal drop of the ramus, preventing contact of any but the buccal cusps on the masticating side, with no contact on the opposite side—the chart showing finally the normal superior short buccal cusps on the same plane as in Dr. Bonwill's diagram, with the longer superior buccal cusps on the lower plane, met by the lower buccal cusp in "balancing occlusion" on one side, notwithstanding the drop of the ramus, on the masticating side the short superior buccal cusp on the plane first shown being met by the long inferior buccal cusp, the long superior lingual cusp on the lower plane being met by the short inferior lingual cusp.

. This articulation of the cusps, which is reproduced in Walker's Physiological Articulator, gives the artificial teeth the cusps essential to the grinding and biting function in mastication, whether with natural or with artificial teeth.

Another diagram farther illustrated the "law of the cusps" in the superior teeth showing the short lingual and the long buccal cusp of the first bicuspid, the nearly level cusps of the second bicuspid and the gradually lengthening lingual and decreasing buccal cusps of one side of the mouth, the reverse condition being found in the inferior molars. He also pointed out the fact that the difference in the planes of the buccal and lingual cusps is really greater in the mouth than appears on holding a tooth out of the mouth with its long axis vertical, as is seen in the usual book illustrations, the inferior molars in the mouth leaning lingually, and the superior molars buccally, increasing the apparent height of the cusps which are raised by their inclination from the vertical, and conversely decreasing the apparent height of the cusps which are lowered.

As the superior lingual and the inferior buccal cusps increase thus in length as we proceed distally, interference of the cusp's lateral movements of the mandible would be inevitable were it not for the drop of the ramus caused by the condyle not only moving *forward* but also gliding *downward* on the slant of the incline of the roof of the glenoid fossa.

The articulation of artificial teeth with cusps indicates the use of an articulator which provides for the downward motion of the condyle and the consequent drop of the ramus preventing interference in the cusps. This, it is claimed, is done by Walker's Physiological Articulator.

DISCUSSION.

In the discussion which followed the reading of this paper, Dr. B. Holly Smith commended the nice, clear distinctions made in minute particulars, and pronounced the views rational, being convinced that the conclusions were correct.

Dr. L. P. Dotterer thought that patients were better satisfied with teeth without cusps, there being less liability of tilting the upper plate, or of knocking about the lower plate.

Dr. J. Y. Crawford said that the paper contained much that was new, and many valuable suggestions. He felt sure that the writer was correct in his description of the true movement of the condyle, and in his conclusions. He was particularly pleased with the prominence given to the upward distal curve, considering that to be an important point in the construction of artificial dentures. He considered a careful study of the articulation of the teeth to be very important, not only in the construction of artificial dentures, but also in giving proper occlusion to fillings. He had not recognized the necessity of cusps for artificial teeth, as much of the food now used can be prepared for swallowing by the mashing process of ordinary dentures. There will always be a difference between the manner of using natural and artificial teeth. The results attained by the writers of the paper have, however, important practical bearings in other directions than in the construction of dentures.

Dr. Dotterer asked Dr. Walker if teeth for full upper and lower plates could be so perfectly articulated as to require no retouching when placed in the mouth?

Dr. Walker replied that as gum sections, though very nicely jointed in investing, may be displaced in vulcanizing, so there was even more liability of plain teeth becoming displaced, necessitating slight touch with the stone, but *in the wax* they could be made perfect, as described by Dr. Dotterer.

In closing the discussion, Dr. Walker said that as insalivation is important and mastication essential to insalivation, cusps were as necessary in artificial as in natural teeth; that artificial teeth could be so articulated with an articulator providing for the drop of the condyle in the movements of the jaw described in the paper.

Subject of Prosthetic Dentistry passed.

There being no other paper on prosthetic dentistry, "Operative Dentistry" was again called, and,

Dr. Gordon White, Nashville, Tenn., read a brief paper on "Capping Pulps."

His method is as follows: The patient first rinses his mouth well with water as hot as can be comfortably borne, to which has been added a little alcohol or a few drops of some antiseptic. The cavity is then washed with warm water and excavated as usual, wiping out occasionally with a pledget of cotton saturated with chloroform. A paper disk is then dipped in chloroform which, after evaporation, leaves the paper sterilized and of its original stiffness. A chloro-percha solution is made having fifty grains of aristol to the ounce of chloroform. A very small portion of this solution is placed on the disk, and the cavity having been wiped out with chloroform, the disk is inverted over the point of exposure and gently pressed to place. Thin cement is flowed over this, and the filling inserted, when the cement has hardened. In over a hundred cases capped in this manner during the past four years, there have been only five failures—two of which were hopeless cases from the start and tried only as an experiment—in one of the others pulp stones were found. In conclusion Dr. White said that he considered capping the dental pulp, when properly performed, to be quite as successful as the average dental operation.

In the discussion of the subject, Dr. McKellops gave a method with which he has had very satisfactory results. The caps cut from asbestos foil, the material being iodoform and glycerine.

Dr. J. Y. Crawford emphasized the importance of differential diagnosis, considering the condition and the age of the patient, of the tooth and of the surrounding tissues. He considers non-sensitiveness of the dentine a pathological signal which must not go unheeded. In some cases, if aseptic pads, as described, are placed over the point of exposure and cement flowed over, the pulp will reassert itself, and through its renewed vitality sensitiveness will be restored to the dentine, but if this is not the case within from twelve to twenty-four hours, the tooth will inevitably give further trouble, sooner or later.

Dr. Boozer considers it important to place one of the balsams over the pad before inserting cement, as the fluid contents of the tubuli—diastase—will decompose the cement and cause the loss of the pulp.

Dr. Jno. S. Thompson cuts the disk from the portion of an envelope containing mucilage—the United States Government using only a pure antiseptic dextrine paste. He moistens the gummed side on the tongue or lip of the patient and applies directly to the point of exposure.

Dr. C. L. Boyd never caps a pulp, if the dentine is non-sensitive above the cornua of the pulp. By observing this rule he has success with ninety out of a hundred cases. He considers aristol very useful in connection with oxide of zinc for this purpose, being very careful not to have any pressure on the cap.

Dr. L. M. Cowardin does not expect success in capping pulps when the pulp is in a pathological condition, for anatomical and histological reasons. In the case of a first permanant molar, exposed before the root is complete, it is very important to make every effort to save the pulp, but after the tooth is fully formed he has little hope that a diseased pulp will ever perform its physiological functions again

Dr. T. M. Allen thinks there is little hope except in case of fresh accidental exposure.

The Association adjourned at this point of the discussion.

Tuesday night a session was held devoted to a stereopticon exhibit and lecture, by Dr. M. H. Cryer, of microscopic slides of sections of the maxillary bones.

Wednesday morning was devoted to clinic.

Wednesday afternoon, Dr. W. T. Arrington, Memphis, Tenn., read a paper entitled "Soft Foil and Conservative Methods of Filling Teeth."

Dr. Arrington's arguments in favor of the use of "Abbey's old-fashioned soft gold" were so convincing, that in the discussion which followed, several expressed a determination to go home and get some and try it at once.

The paper and the subject was discussed by Drs. Beadles, G. F. S. Wright, L. G. Noel, and T. M. Allen, until the time arrived which had been set apart for the discussion of the points recommended for discussion by the committee.

The subject of the appointment of a committee of five to confer with a similar committee from the American Association in regard to a proposed consolidation of the two Associations—Southern and American—forming a National Association, brought out the expression of a strong sentiment of disapproval of the proposition. After some discussion it was agreed to appoint the committee asked for, further discussion to be postponed until after a report from the joint committee, no definite proposition having been submitted.

Drs. L. G. Noel, J. T. Calvert, Fr. Peabody, E. T. Beadles and J. R. Knapp, were appointed on the committee.

The subject of "Operative Dentistry" was again opened for discussion.

Dr. Crawford spoke of the experiments of Dr. W. D. Miller in testing the antiseptic qualities of various filling materials, only those containing copper, and Abbey's soft gold, *which had not been exposed to heat*, standing the culture test. Exposure to red heat seems to destroy some subtle quality, "a something," which gives it antiseptic qualities, so that sections of old teeth filled by Maynard, Badger, etc., thirty and forty years ago, still retain the antiseptic influence of Abbey's soft gold.

This appeared paradoxical to Dr. Peabody who quoted the Good Book as to "purifying by fire."

Dr. W. E. Walker referred to the paper of Dr. Gordon White, and spoke of two varieties of non-sensitive dentine found in cases of mass pulp-exposure. In one case the dentine will be of more than normal hardness, apparently due to infiltration with lime salts—a not unfavorable condition. In the other variety the dentine will vary in color from nearly normal to a dark gray, and will be found very soft under the instruments, and the tubuli filled with decomposing matter. On reaching the pulp there will usually be found what might be termed a pocket of pus and gases. In cases of this class hot applications cause pain through the expansion of the gases and consequent pressure upon the living portion of the pulp. He spoke of the division suggested by Dr. Harlan, of subjective and objective stages. These varying conditions illustrate the importance of differential diagnosis. Dr. Walker also spoke of the importance of a knowledge of temperament in diagnosis for pulp treatment; the value of studying the patient as a whole, habits of life, occupation, etc. Persons of the nervous temperament are much better subjects for pulp-capping than those of the lymphatic temperament, the latter, on the other hand, being better subjects for pulp devitalization.

Dr. Noel and Dr. Peabody spoke of the value of copper amalgam in filling children's teeth, its antiseptic qualities in these cases more than balancing its lack of durability.

Dr. R. C. Young asked for advice in dealing with devitalized deciduous teeth, in order to retain them until the proper time for them to be lost.

Dr. Walker said that he had very favorable results from filling them, as recommended by Dr. Cravens some years ago, with phosphate of lime, cleaning the cavity and the root canals as thoroughly as possible, in dealing with very young patients, taking the usual antiseptic precautions, filling the root canals with phosphate of lime in the magma state, working into the soft mass all the dry phosphate that it will take up, and filling the cavity with cement, gutta percha or amalgam as desired. This root-canal filling offers no obstacle to the incoming permanent tooth.

Dr. J. Y. Crawford emphasized the importance of keeping a devitalized tooth, after filling, non-antagonized, by grinding off the morsal surface at intervals as needed, until the bifurcation of the roots is reached, supplementing absorption by exfoliation.

The subject of pulp capping was further discussed.

Dr. S. E. C. Watkins would make very careful discrimination in the choice of cases, but would cap all freshly-exposed pulps. He spoke of the advantages of oil of cloves over creosote, as not coagulating the surface of the exposed pulp.

Dr. R. D. Adair thinks it not justifiable to destroy a living pulp. He would give all but very desperate cases a chance to live by capping; if they die the tooth is no worse off than if the pulp had been killed in the beginning, and there was the chance that it might perhaps live.

Dr. Arrington closed the discussion on Operative Dentistry and the Association adjourned.

The Association was called together again at 7:45 Wednesday night.

### DENTAL EDUCATION.

Dr. W. E. Walker, Pass Christian, Miss., read a paper entitled "Ought the Dentist to be a Graduate in Medicine?"

Answering the question in the affirmative, Dr. Walker brought forward an array of arguments sustained by quotations from the dental journals. The first point made was that in order to save diseased teeth it is necessary to put the surrounding tissues in a healthy condition, as thorough a knowledge of medicine being necessary for the treatment of the oral tissues, as for the treatment of the eye, the ear, or the nose. As the ophthalmologist, the otologist and rhinologist must all be medical graduates, why not the stomatologist also?

Again, in the treatment of patients for oral and dental disorders, it is frequently necessary to administer, both internally and hypodermically, medicaments of which we would have a much more thorough knowledge in their effects upon the general system after a full medical course than we have after a course in dental *materia medica*, and dental therapeutics. A knowledge of temperaments, temperature, pulse, etc., is as essential to the stomatologist as to other specialists in medicine.

He cited cases both from the dental journals and from his own practice, showing the necessity for this knowledge of medical science in dental practice.

Admitting that the M. D. degree is of importance to the D. D. S., both for the medical education it implies and for the legal protection it affords, his next question was: "When shall this degree be obtained? After the D. D. S., before the D.D.S. or simultaneously?"

Experience has shown that the medically-trained man does not usually make a skilled practical dentist for the reason among others that his manipulative faculties have been neglected until too late in life.

As the skilled pianist begins very early with finger-exercises, so should the dentist be early trained in the acquirement of that nicety of touch—that manipulative ability so essential to the skillful dentist. The ideal course would be the simultaneous course of medical and dental education, with time set apart for the cultivation of the special faculties of the practical dentist. Such a course should entitle to the combined M.D.-D.D.S. degrees, for which DR. WALKER suggests a new degree, S.D., *Stomatologiæ Doctor*—doctor of stomatology—a degree which would imply more than M.D., more than D.D.S., it would imply both in one—the knowledge of the M.D. and the skill of the D.D.S., necessary for the mouth doctor. It would be a degree which should give the medical and the legal standing of the other medical specialists—the ophthalmologist, the otologist, the rhinologist, the stomatologist, all standing on a professional, scientific and legal plane of equality.

In the discussion of the subject DR. S. W. FOSTER agreed with the essayist that dentists have need of a broader medical education, especially on the line of diagnosis. He considered the suggested new degree a step in the upbuilding of the profession on a line hitherto unsuggested.

DR. W. W. WESTMORELAND and DR. T. M. ALLEN believe that the curriculum of the dental college of to-day provides all that is needed by the dentist. If the student is capable of passing the State Examining Boards he is prepared for anything he may encounter in his practice. DR. ALLEN thinks the effort to acquire both the M.D. and the D.D.S. would result in a mere smattering of medicine and dentistry with no efficiency in either.

DR. B. HOLLY SMITH said that the medical colleges are ad-admitting more and more generally the importance of a knowledge of the principles of dentistry to the general physician, and are making every effort to have medical students taught something of dental science. He considers the degree D.D.S. conferred after a course in the dental college of to-day, evidence of ample training in medical science to satisfy the most exacting demands. Lecturers in the dental colleges have to keep up with the trend of advanced thought, working constantly for higher attainments.

DR. B. H. SMITH thinks that a man with a thorough dental education would make a better physician than the most thorough medical education could make of him a dentist.

DR. S. W. FOSTER spoke of a too common difference existing between medical and dental therapeutics—the latter being largely empirical, based on statements read in journals and heard in discussion. Under certain conditions some one claims to have obtained good results from the use of a certain drug. Straightway when we encounter apparently the same conditions, we apply the same treatment—not based upon a knowledge of rational therapeutics, or a knowledge of cause and effect, but on the say-so of some one else. As dentists we should know more of medicine.

DR. H. E. BEACH protested against the oft-repeated statement that medicine is a science. There is but one science and that is mathematics. When medicine can say with mathematical precision that a certain line of treatment will have a certain result, then it will be scientific. If medicine were truly a science every physician in the world would prescribe the same treatment under the same conditions, always with the same results. Medicine as practiced to-day is and must be largely empirical.

DR. HURING thinks the paper read should inaugurate a dental revolution because of its far-reaching conclusions. The words dental education are misapplied. Dental education does not include all that the student should know before he is fit to come up for matriculation before a Peabody or a Crawford—a poor ignorant boy who has just left the plow-handles, is not ready to begin his dental education—he requires a preliminary education. There are too many dental colleges; too great a demand for students, not for the good they can be trained to do their fellowmen, but for the money they bring in. If every student were compelled to prove his fitness for matriculation, and only those really ready for a college course admitted, the degree of D.D.S. would be a prize worth striving for—an honor to its possessor. Under the present system every student expects his diploma, and there is no reason why he should not expect it.

Dr. T. C. West, Natchez, Miss., read a paper on "Dental Education."

His paper was a plea for preliminary education as a prerequisite for matriculation in the dental college—a strong and broad foundation on which to erect the edifice of learning. The first year in the dental college is often spent in the vain endeavor to grasp the situation—in learning how to learn. His mental faculties have not been developed and trained—not before the second year is he able to digest the mental pabulum offered by the college lecturers. At the end of the third course he may have struggled through and received his diploma, but when he goes before his State Board he fails to come up to the required standard. We say to the college: "Raise your standard to meet the requirements of the law," but the college should say: "Raise your boys to the proper standard before sending them to us."

In the discussion of this paper Dr. B. Holly Smith spoke of the value of manual training, and especially of the cultivation of the left hand as being of very great advantage to the dentist. There would be a much more general use of the left hand but for a mistaken prejudice in the training of children.

Dr. V. E. Turner spoke of the lack of thoroughness in the mental training and discipline of the present system of education. A man devotes himself to what he understands the most readily and with the least study or effort, and keeps to that without being well grounded in the text-books. The college affords every opportunity, but they do not enforce the full use of the opportunities offered.

Dr. M. C. Marshall, St. Louis, read a paper entitled "Broader Education."

He said that though the standard of dental education has been constantly elevated, yet the American idea of "get there" rushes many young men into practice long before they are qualified to enter upon the duties of a scientific vocation. He referred to the discussion in the American Dental Association on the increasing number of dental colleges and the number of dental graduates, the supply threatening to exceed the demand, and the best methods of arresting this influx into already too plethoric ranks.

Dr. Marshall suggested that dental education be made broader and the field of operations enlarged. Especially is the care of children in the various disturbances arising from pathological dentition properly belong to the dentist. To this end the dentist should be taught more medicine. He should also be better prepared to administer anæsthetics—the treatment of facial neuralgia—inflammatory conditions of the oral mucous membranes, pathological conditions of the glands, tumors of whatever character, in fact, all conditions of the mouth requiring medical, surgical or mechanical interference. This is the domain of the dentist, and not until we are prepared to enter upon it and scientifically discharge the duties requisite thereto will we be dentists in the full acceptance of the name. It is true that there are already a few practicing along these lines. These are the pioneers preparing the way for our legitimate occupancy.

The subject of Dental Education was further discussed by Drs. J. Y. Crawford, Fr. Peabody and Arrington, Drs. Walker, West and Marshall closing the discussion of their respective papers. Subject passed.

### DENTAL HYGEINE.

Dr. R. G. Young, Anniston, Ala., read a paper entitled: "Can the Dentine be Reached Through Systemic Treatment?"

Accepting as a fact that the teeth do undergo structural changes, teeth that at one time are so dense as almost to defy the keenest temper of an instrument, at a later period appearing soft and chalky, and this without any apparent derangement of the general system, though often associated with close mental application or with undue yielding to the demands of fashionable society, he concludes that the changed character of the tooth structure is due to abnormal elimination of lime salts, as proved by urine analysis. The teeth (and probably the bones, also, though these are not so open to examination), being thus deprived of their normal supply of nourishment, the effect is seen in the changed character of the dentine. A return to hygienic methods of life is not sufficient, as under the conditions named the appetite is too poor to relish the diet which offers the necessary elements of tooth structure--brown bread, the cereals, etc. It is like offering a pint of some decoction or infusion when a grain of the alcoloid of the same principle would give better results. Diet must be supplemented by the direct administration of the lime salts, especially the syrup of the hypophosphites, from which he is convinced great benefits many be derived.

Discussion of the subject was postponed to Thursday night, Thursday morning having been set apart for clinics, and Thursday afternoon to an old-fashioned Georgia barbecue, tendered to the Association by the 'Cue Club of Atlanta.

The Association was called to order again at 7:30 P.M. Thursday.

In the discussion of Dental Hygeine, DR. J. Y. CRAWFORD emphasized the importance of cleanliness of the mouth and teeth, and said that the first operation for every patient should be a thorough cleaning.

Proper information given on this point has great moral force and far-reaching in its results. He dwelt particularly upon the importance of cleanliness of the mouth, and the eradication of all disease previous to the eruption of the permanent teeth. The baby teeth should not be allowed to afford lodgment to septic matter to produce decay of the permanent teeth.

DR. T. C. WEST considers that the greatest difficulty encountered is in getting hold of the children early enough. Parents will not bring them until they are actually suffering from toothache.

He thinks the care of the teeth should be made one of the subjects of school instruction, beginning in the kindergarten.

Clean teeth should be made a matter of even more importance than clean faces and hands and finger-nails. If inspection and catechism along this line was begun in the kindergarten, immense good would result.

Dr. R. C. Young spoke of the good effect produced upon patients by habitually making a litmus paper test of the secretions, putting them upon a regime of Phillip's Milk of Magnesia for a few weeks and showing them the results. Such little things cause them to appreciate more fully the value of dentistry.

Dr Cowardin, while admitting that there may be a hardening through the deposition of calcic salts, does not believe there can be any retrograde metamorphosis as there is no circulation through the dentine structure. He agrees with Dr. West that we cannot get hold of the children early enough. Departure from normal diet begins very early and we cannot expect normal tissues with abnormal and unhygienic habits of life, often, almost, from birth.

### HISTOLOGY AND MICROSCOPY.

Dr. J. H. Boozer, Atlanta, Ga., read a paper on "The Contents of the Dentinal Tubuli, the Action of Medicaments, the Preservation of the Teeth by Fillings, the Phenomena of Pain."

He said that while not a microscopist himself, he had studied closely the works of authorities along the lines named, and was forced to believe that the microscope was chiefly valuable in proving conclusions already reached through microscopic study. Different men see very different things *and illustrate them*, and draw very different conclusions from the same microscopic slides. The arguments of each appear unanswerable when studied separately, but taken together they make a sorely-trying muddle, producing confusion and discouragement.

The paper showed study of the writings of Tomes, Black, Sudduth, Boedecker, Trueman, Harlan, Miller, and other eminent authorities, from all of which the author deduced theories of his own as to the contents of the tubuli, the growth and nourishment of the teeth, calcification and decalcification, the action of medicines, the preservation or loss of teeth under different fillings, etc.

The paper was briefly discussed by Drs. Young, Wright, and Crawford and the subject passed.

On recommendation of the Executive Committee, Dr. Southall Dixon, Bolivar, Tenn., and Dr. S. C. G. Watkins, Mont Clair, N. J., were elected to active membership.

No report being offered by the Committee on Chemistry, the subject was opened for discussion.

Dr. W. E. Walker said that as no place was offered on the program for Incidents of Office Practice, he would cite a case on which he would like to have an expression of opinion, and which might come under the head of Chemistry, caries being to a certain extent a chemical process.

The case was that of a young lady, quite young, whose teeth had been under his care for a number of years and which were of excellent quality and kept in perfect condition until the present season, when, on her return home from school in a large city, after an absence of only a few months, he found thirty-nine cavities of decay, the teeth literally melting away. He had made diligent inquiry but could not learn that there had been any serious errors in diet, no "midnight suppers"—that bane of the girl's boarding-school. He could see no cause for the very radical change in the character of the tooth structure unless, perhaps, the unusual confinement and perhaps some mental strain.

Dr. S. C. G. Watkins thought that nervous strain and other remote causes should be taken into consideration in such cases.

Dr. Geo. H. Winkler has recently published a paper giving the remarkable results following the administration of *creosotum*, the homeopathic preparation, in just such cases. In one case where thirty or forty cavities of decay had been developed in a short time; after the administration of this remedy, in the same length of time and under the same circumstances, only two or three new cavities had developed. When the gums are hypersensitive, the saliva ropy, etc., *creosotum* will have the happiest effects, the mouth becoming as sweet and clean as that of a new-born babe.

Dr. B. H. Smith mentioned the fact that individuals coming to Florida from California, Maine, and other remote points, said that their teeth decay very rapidly. He has been unable to ascertain the cause of this, which, however, is a fact that cannot be disputed.

Dr. C. L. Boyd had seen similar cases though not so extreme. There must be some local cause other than change of locality or diet. Subject passed.

The committee on Voluntary Essays offered a paper from Dr. W. H. H. Thackston––a report upon the professional literature of the past year—" the crop and vintage of 1894–5." He spoke of the superabundant supply of monthly and quarterly publications as being a tax upon the time, patience and pocket, many of them being largely filled with " thrashed-over straw," but in nearly all may be found some practical hint, some invention or discovery, or some original application of the laws and principles that underlie dental science. Among the annual publications he spoke of Catching's Compendium as being of the highest value for ready reference to all that is new or needful. He commends the annual reports of the different dental associations to all who desire to keep abreast with the progress of dental science, the most important contribution to our literature being the Transactions of the World's Columbian Dental Congress, as marking an epoch in our history. The paper embraced a brief review of the most noted volumes of the year, and concluded with a sketch of the noted progress and advancement made in the character of our literature and of our professional attainments.

Dr. Francis Peabody read a brief paper describing two cases in orthodontia of which casts were presented for examination.

Dr. A. M. Scott, of New York, read a voluntary essay on the "Treatment of Oral Acidity; Local and Systemic." The conditions existing when the reaction of the oral fluids are abnormally acid, as from nutritional disturbances, abnormal systemic conditions, functional perversion, threatens the integrity of tooth structure and associate parts—erosion, hyperæsthemia of tooth structure, chronic inflammation of the gingival margins of the gums, recession of the gums, etc. In all of these cases a non-corrosive alkali is naturally suggested, chalk. calcined magnesia, bicarbonate of soda, lime-water, etc., have all been used with more or less indifferent results. Their action is only transient; they are gritty and insoluble; they are anything but pleasant to the taste.

To meet both these conditions and overcome the objections to the agents named, magnesium hydrate offers all the advantages possible. And there is but one form suitable to the purpose and that is Phillip's Milk of Magnesia, a powerful antacid, chemically pure, odorless, tasteless, free from grit, not subject to precipitation and containing neither gum, starch nor glycerine—simply water and magnesia.

The association adj urned after the reading of these voluntary essays which were accepted and passed without discussion.

Called to order at 9:15 Friday morning.

The Chair: The Committee on Appliances and Inventions not having prepared any report, any one having anything new which has not been presented to the association, has the privilege of the floor.

Dr. C. D. Carpenter described and exhibited the appliances used in the correction of irregularities in several cases, of which the models were passed around. He also passed around for examination a tooth containing a large amalgam filling which had done service in the mouth for fifty-three years.

Dr. W. E. Walker exhibited and explained his Physiólogical Articulator, an articulator in which the part representing the condyle is placed at an adjustable angle with the plane of articulation, a new feature in a dental articulator, and one found to be necessary in reproducing the articulation of models of the natural teeth, the average angle having been found to be about 35°, but ranging from 22½° to 45°, in models taken from a large number of patients; the former being a case of extreme irregularity, the latter one of abnormal bicuspid cusps. The articulator is provided with a degree gauge for registering the angle in each case, and a millimeter gauge for registering the correction of a wrong bite. These figures being recorded upon the plaster models of any given case, they can be laid aside for future use, and the articulator used for different cases.

The Publication Committee reported a proposition from the S. S. White Manfg. Co. for the publication of the proceedings of the present meeting in the *Cosmos*, and in book form, free of expense to the association. The proposition was accepted.

DR. B. H. CATCHING read the report of the Committee on Revision of the Constitution and By-Laws. The proposed amendments were read by sections and discussed. The final revision was adopted, as a whole, and on motion of Dr. Crawford, ordered printed with the proceedings of the present meeting.

The Committee on Clinics presented a report embracing a clinic by Dr. W. H. Richards, Knoxville, Tenn., demonstrating his method of loading lower rubber plates with low fusible metal either for the purpose of making a new plate for a much absorbed gum stable by additional weight, or by restoring an old plate to comfortable service cheaply, or for prolonging the use of a temporary plate under which absorption has gone on rapidly, but not sufficiently for a permanent plate.

DR. W. T. ARRINGTON, Memphis, Tenn., made a clinic on molar proximal and crown compound cavity, using Abbey's No. 5 soft gold with No. 4 tin foil, the foils being folded in a ribbon with alternate layers of tin and gold.

DR. L. E. CUSTER, Dayton, Ohio, fused porcelain in his electric oven, which does away with all odor, noise, gas and heat in the room.

DR. L. G. NOEL had a series of clinics with children treating almost every variety of disease incident to childhood.

DR. W. B. FINNEY demonstrated a double contour of the central incisor extending across the cutting edge, the contour built down to natural shape of the tooth, and done with instruments of his own design.

DR. C. L. ALEXANDER, Charlotte, N. C., demonstrated his suspension denture, and exhibited a disk mandrill of his own invention.

Dr. W. E. Walker, Pass Christian, Miss., exhibited three forms of his new Physiological Articulator and the well-known Bonwill Anatomical Articulator demonstrating with casts of the natural teeth that in Bonwill's Anatomical Articulator, while they will *occlude*, they will not *articulate* so as to permit the functional movements of "grinding" and "biting." In the position for "biting" the molars come in contact before the morsal surfaces of the incisors can engage. In the position for "grinding" the long superior lingual cusps touch the long inferior buccal cusps on the side from which the jaw is receding, preventing contact on the opposite side. The models of natural teeth on the same "loops" taken out of Bonwill's Articulator after the above demonstration, and placed in Walker's Physiological Articulator, both occlude and articulate perfectly in any position in which the lower jaw may be placed, that can be taken by the natural jaw, the "biting" and "grinding" articulation being reproduced as in the mouth from which the casts were taken.

In the Bonwill Articulator the part representing the glenoid fossa is placed at right angles to the part representing the ramus, thus forcing the part representing the condyle to move horizontally forward with no accommodation for the long upper lingual and the long lower buccal cusps in the "grinding" and "biting" positions.

In the Walker Physiological Articulator the part representing the glenoid fossa is placed on an incline at a variable angle corresponding to the variations found in the roof of the glenoid fossa in different individuals, thus reproducing the movement of the mandible and the articulation of the teeth in the different positions which it takes in the subject.

Dr. M. G. Marshall, St. Louis, Mo., presented a new feature in bridge-work—a gold box frame having cavities into which the porcelain teeth are fitted and so grasped by the gold that they cannot be pressed out—they are further retained by means of oxyphosphate of zinc or melted sulphur. In case of fracture a tooth can be replaced without removing the bridge, and no gold-grinding surface is necessaary.

Dr. P. W. Onderdonk, New York, demonstrated the attachment of a Logan crown by means of a gutta-percha washer, the roof end being smeared with a varnish composed of resin and chloroform.

Dr. E. P. Beadles, Danville, Va., made a combination filling with cohesive and non-cohesive gold, using hand pressure entirely, cavity shaped without retaining points. He maintains a perfectly erect position in all his operations and never has a backache.

The report of the chairman of the Committee on Operative Dentistry and a paper from Dr. D. L. Boozer, Columbia, S. C., entitled, "Public Opinion vs. Dentistry," received too late for reading, were ordered included in the Transactions.

Dr. J. Y. Crawford read a series of communications from the Nashville Chamber of Commerce, the Mayor, the City Council, the Board of Public Works and the officials of the Tennessee Centennial tending a most cordial invitation to hold the annual meeting of 1896 in Nashville during the Centennial Celebration.

Nashville was accordingly selected as the next place of meeting, and the association adjourned till November, 1896.

